# Comparison of Rapid Rehabilitation after Anterior Cruciate Ligament Reconstruction with Tensioning Technique and Traditional Rehabilitation

**DOI:** 10.1155/2022/6779207

**Published:** 2022-07-01

**Authors:** Tianfu Jin, Yanlin Li, Guiran Yang, Xinyu Liao, Guoliang Wang, Fuke Wang

**Affiliations:** Department of Sports Medicine, The First Affiliated Hospital of Kunming Medical University, Yunnan 650032, China

## Abstract

To investigate the efficacy of a fast rehabilitation program for the recovery of knee joint function after arthroscopic autologous hamstring tendon transplantation for reconstruction of the anterior cruciate ligament (ACL), from January 1, 2017, to March 31, 2019, a total of 65 patients with ACL injury were randomly divided into a study group and a control group. Both groups were treated with autologous hamstring tendon to reconstruct the anterior cruciate ligament, arthroscopic transplantation, and decompression techniques. The research group was treated with a fast rehabilitation program. The control group was treated with traditional rehabilitation program. Knee flexion angles were measured at 2, 4, and 8 weeks postoperatively. KT-1000 knee anterior stability was measured at 3, 6, and 12 months after operation. Knee function was assessed by subjective knee function assessment scale (IKDC) and Lysholm knee score. The knee curvature, KT-1000 measurement, IKDC score, and Lysholm score were compared between the two groups before and after treatment. KT-1000 measured value, IKDC score, and Lysholm score in 2 groups were significantly improved 3, 6, and 12 months compared with those before treatment, and the difference was statistically significant (*P* < 0.001). Comparison between the two groups: 2 weeks, 4 weeks, and 8 weeks after treatment, the knee curvature in the study group was better than that in the control group, and the difference was statistically significant (*P* < 0.001); there was no significant difference in the measured values of KT-1000 between the two groups 3, 6, and 12 months after treatment (*P* > 0.05); IKDC score and Lysholm score in the study group 3 and 6 months after treatment were significantly better than those in the control group, with statistical significance (*P* < 0.001); there was no significant difference in IKDC score and Lysholm score between the two groups 12 months after treatment (P >0.05). Autograft hamstring tendon transplantation and tense-reducing technique for anatomical reconstruction of anterior cruciate ligament under arthroscopy combined with rapid rehabilitation program can quickly, safely, and effectively restore the knee function of patients, greatly shortening the rehabilitation period of patients.

## 1. Introduction

The anterior cruciate ligament (ACL) is the main factor to maintain the stability of the knee joint, and its injury is one of the more serious sports injuries in clinical practice. Arthroscopic ACL reconstruction is the first choice for the treatment of ACL fractures [1]. In the early stage, our department innovatively applied the self-created “triangle” braided tensioning line tensioning technique for patients undergoing arthroscopic anatomical reconstruction of ACL with autologous hamstring tendon transplantation. To a certain extent, the problems of graft relaxation and bone marrow zone enlargement caused by “wiper effect” and “bungee effect” after treatment were solved [2, 3]. A reasonable postoperative rehabilitation training program is also crucial to the recovery of knee function [4]. At present, there is no unified standard for rehabilitation training after ACL reconstruction; therefore, this study provided reference for the rehabilitation of patients after anterior cruciate ligament reconstruction by retrospectively analyzing whether the rapid rehabilitation program can ensure the safe and rapid recovery of knee function in patients with anterior cruciate ligament reconstruction under the condition of graft fixation.

## 2. Data and Method

### 2.1. Inclusion Criterion and Exclusion Criterion

Inclusion criteria for this study: (1) patients diagnosed with ACL injury or fracture undergoing anatomical reconstruction with ACL tensioning techniques; (2) patients aged 19-45 years, male or female; (2) physically healthy and without patients with other medical history or joint deformities; (4) patients without severe meniscus or cartilage damage; and (5) patients with complete follow-up. Exclusion criteria: (1) patients with multiple ligament injuries in the knee joint, (2) patients with knee osteoarthritis, (3) patients with joint infection, and (4) patients with incomplete follow-up.

### 2.2. General Data

A total of 65 patients with ACL fractures were admitted to our hospital from January 2017 to March 2019, and all patients met the inclusion criteria. The 65 patients were divided into two groups, the study group and the control group, by a random distribution table. These patients underwent anatomical reconstruction of the ACL using the “triangle” braided tension wire tension technique. There were 33 patients in the research group, including 19 males and 14 females, with an average age of 29.06 ± 6.96 years and an average BMI of 22.67 ± 2.51 kg/m^2^. There were 18 left knee joints and 15 right knee joints. The time from injury to operation was 6.70 ± 3.82 months. There were 32 cases in the control group, including 20 males and 12 females, with an average age of 29.09 ± 6.48 years and an average BMI of 22.88 ± 1.96 kg/m^2^. 18 cases are on the left knee and right knee in 14 cases. The mean time from injury to operation was 6.53 ± 3.91 months. This study was approved by the hospital ethics committee, and all patients signed informed consent. There was no statistical difference between the two groups in preoperative age, gender, affected side, BMI, complication of injuries, and the time from injury to operation (see Tables 1 and 2). “Triangle” braided tensioning line tensioning technique was used for anatomical reconstruction of ACL in both groups, which was done by the same team of surgeons. The study group was given postoperative rapid rehabilitation training. The control group was given postoperative traditional rehabilitation training.

### 2.3. Operation Method

**Figure 1:**
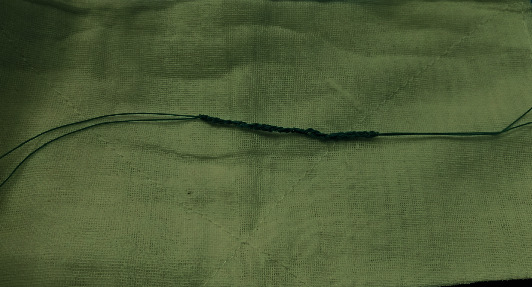
Braided tension line.

**Figure 2:**
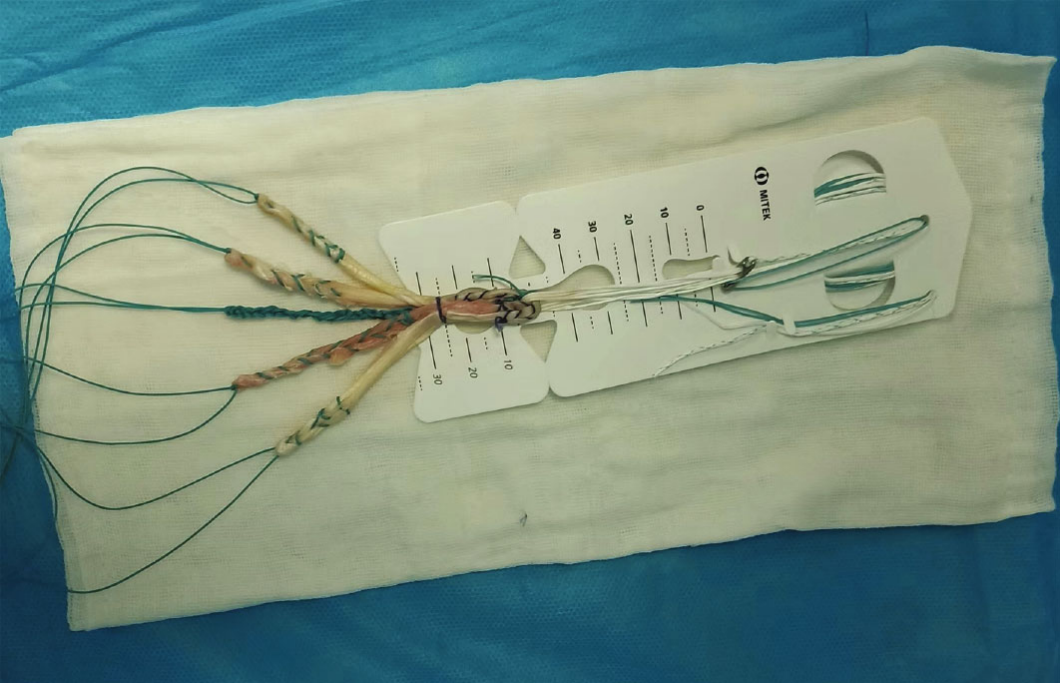
Braided graft.

**Figure 3:**
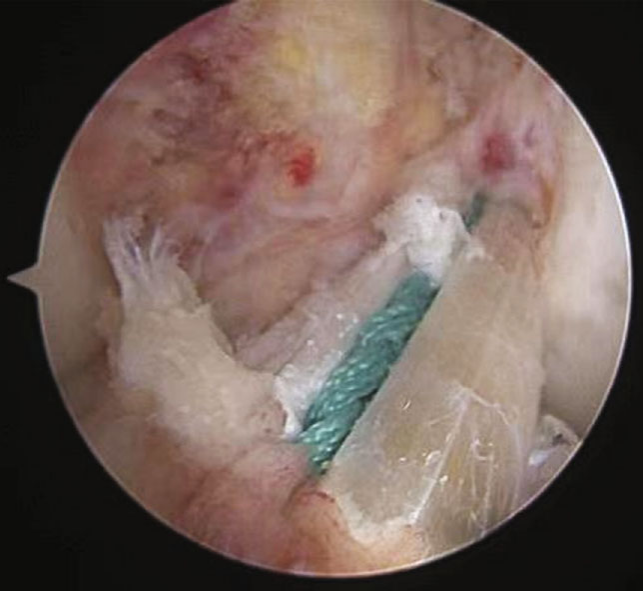
The reconstructed anterior cruciate ligament is seen under arthroscopy, with the tensioning line wrapped around the graft.

Patients in both groups underwent arthroscopic reconstruction of the anterior cruciate ligament with autogenous hamstring tendon and single bundle under combined spinal-epidural anesthesia. Arthroscopic exploration was conducted for determination the injury of the ACL. Then, oblique incision was made at 1 cm inwards of the tibial tubercle and semitendinosus and gracilis muscle were removed. Both ends were braided 3-4 cm with no. 2 ETHIBOND nonabsorbable suture and then were folded in half into 4 strands, about 9 cm long. Two strands of “triangle” braided tensioning line created by our department were added into the graft tendon (ETHIBOND suture, Figure 1), with about 9 cm long. One end of the tensioning line was knotted and fixed at the half fold of the graft tendon. No. 2 ETHIBOND nonabsorbable suture was used at the split end of the graft tendon (Figure 2). Tibia and femur tunnels were made with tibia locator and femur locator, respectively. Graft was pulled into the bone tunnel, and the end of the femur was fixed with a cross screw. The tibial end was fixed with absorbable interfacial screw combined with homemade portal nail after 20 passive flexion activities of the knee joint (Figure 3). Lachman test and front drawer test were negative. No ACL impingement was observed under the microscope during knee flexion and extension. Intraoperative injection of dexamethasone, ropivacaine, saline mixture, and tranexamic acid was used to prevent postoperative pain and bleeding. No drainage tube was placed during the operation, and the operation ended [2, 5, 6].

### 2.4. Rehabilitation Methods

**Figure 4:**
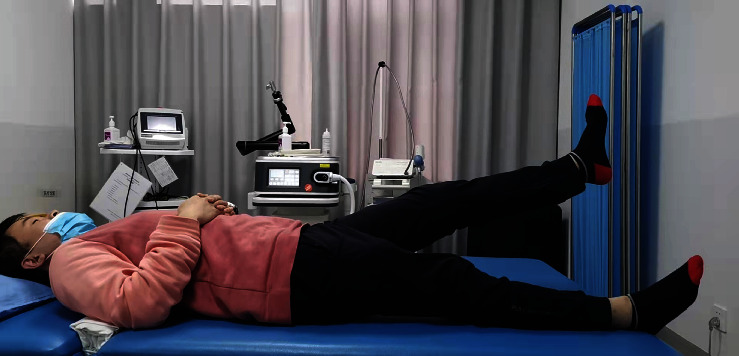
Straight leg elevation for quadriceps training after the operation.

**Figure 5:**
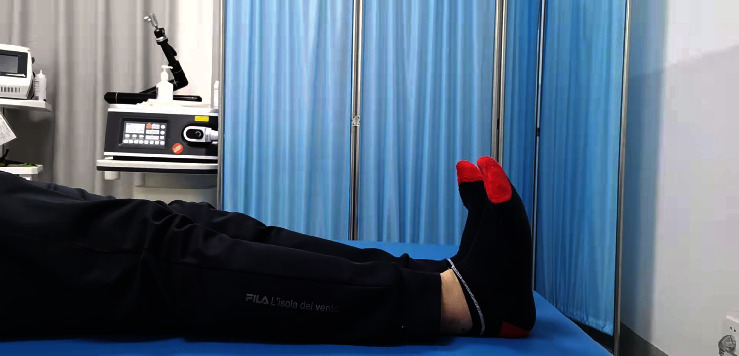
Ankle pump training after the operation.

**Figure 6:**
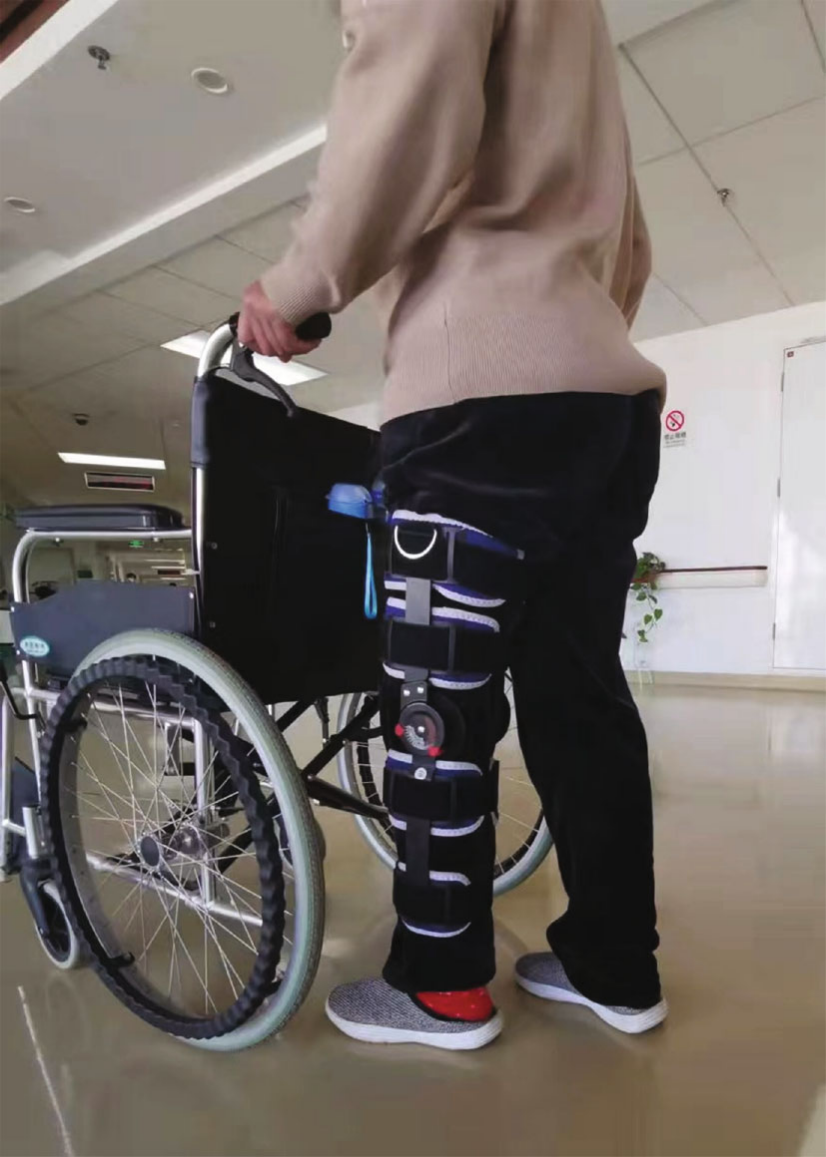
Walk with partial weight bearing under the protection of a hinged brace after the operation.

**Figure 7:**
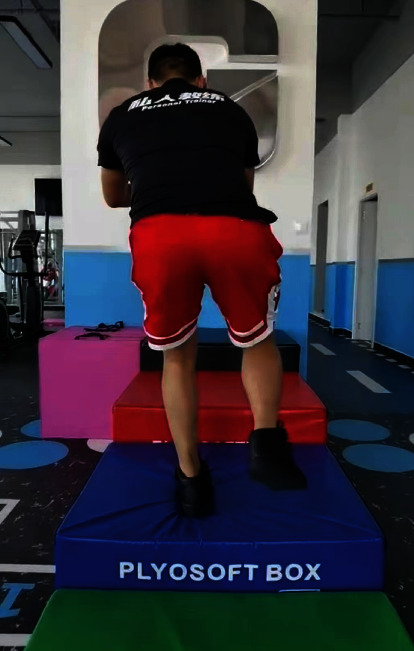
Balance training after the operation.

Rapid rehabilitation in the study group: the patients started the quadriceps femoris and gluteus isometric contraction (Figure 4) and “ankle pump” training (Figure 5) as soon as they were awake from anesthesia on the day after operation, as well as passive patella exercises. The first day after the operation, the passive knee bend was started, and the knee bend reached 90° after 7 days and was close to normal after 8 weeks. The patients walked with partial weight bearing under the protection of a hinged brace and with pain tolerance the third day after operation (Figure 6). Two weeks after the operation, the patients could walk with full weight bearing on crutches with pain tolerance. Eight weeks later, the muscle strength of the affected lower limb was enhanced, and the knee joint stability training was performed (Figure 7) to restore the basic functional activities of the affected lower limb, improve the cardiopulmonary function, and improve the muscle strength of other key areas. Ten weeks after the operation, running training and one-leg jumping training were started. Three months after the operation, the patients began moderate physical activities of their own preference. Six months after the operation, the patients began complete strenuous exercise (activity level, weight bearing, and brace use; see Table 3).

Conservative rehabilitation in the control group: after the operation, the patients started quadriceps femoris, gluteus isometric contraction, and “ankle pump” training, as well as passive patellar exercise. The patients were fixed in the straight position with a brace for one week. At week 2 after the operation, the patients started a small range of passive knee movement. At week 4, the patients' knee bend could reach 90°, at week 8, the knee bend could reach 120°, and at week 12, the knee bend was close to normal.

Two weeks after the operation, the patients walked with partial weight bearing using double crutches under the protection of a hinged brace. Eight weeks after the operation, the patients walked with full weight bearing without crutches. At week 12, brace protection was removed, and normal gait was restored. After 12 weeks, knee terminal extension exercise, balance exercise, and muscle strength exercise were performed to restore the basic functional activities of the affected lower limbs. Three to six months after the operation, the patients started running training, flexibility exercises, cardiopulmonary function improvement, muscle strength improvement in key areas of the body, and proprioception exercises. Six months after the operation, the patients began moderate physical activities of their own preference. Nine months after the operation, the patients began complete strenuous exercise (activity level, weight bearing, and brace use; see Table 3).

### 2.5. Evaluation Indicators

The knee flexion degrees of the two groups were measured at 2, 4, and 8 weeks after the operation. Anterior stability of the knee joint was measured at 3, 6, and 12 months postoperatively [4]. The International Knee Documentation Committee (IKDC) [7] and the Lysholm knee score are used for functional access to the knee [8]. The knee flexion, KT-1000 measurement value, IKDC score, and Lysholm score were compared between the two groups before and after surgery. The final follow-up time point was 12 months after surgery.

### 2.6. Statistical Analysis

Statistical analysis was performed using SPSS version 19.0 statistical software (IBM, USA). The measurement data of the patients in the study group and the control group conformed to a normal distribution. The differences between the two groups were analyzed by the *T* test of two independent samples, and the count data were compared by the *χ*^2^ test. Repeated measures analysis of variance was used to compare the measurement data at preoperative and postoperative follow-up time points in the same group, and SNK-q test was used for pairwise comparison. Patient age, BMI, time from injury to surgery, knee flexion, KT-1000, IKDC score, and Lysholm knee score were all with normal data distribution. Two-sided *P* < 0.05 was considered statistically significant.

## 3. Results

There were no intra-articular infection, deep vein thrombosis, vascular and nerve injury, and other complications in the two groups after operation. Anterior drawer test and Lachman test were both negative. All patients were fully followed up (11-14 months, mean 12 months). Active and passive movement of the affected knee is not restricted. Repeat MRI showed good healing of the ligament and bone tunnel. When the tibial internal fixator was removed after the patient returned to the hospital, a second arthroscopy was performed, which showed that the reconstructed ligament had good continuity and the synovium and other tissues were well covered.

KT-1000 measurement values (Table 4), IKDC score (Table 5), and Lysholm score (Table 6) of patients in 2 groups at 3, 6, and 12 months after the operation were significantly improved compared with those before the operation, and the differences were statistically significant (*P* < 0.001). The knee flexion angle in the study group 2 weeks after the operation (88.48 ± 9.35), 4 weeks after the operation (101.36 ± 6.23), and 8 weeks after the operation (125.85 ± 10.29) was better than that in the control group 2 weeks after the operation (65.03 ± 4.03), 4 weeks after the operation (91.56 ± 8.41), and 8 weeks after the operation (114.34 ± 11.81) (Table 7); the difference was statistically significant (*P* < 0.001). Three, 6, and 12 months after the operation, the KT-1000 measuring value (2.03 ± 0.18, 2.03 ± 0.18, and 2.13 ± 0.42) in the study group was comparable to that in the control group (2.03 ± 0.18, 2.03 ± 0.18, and 2.13 ± 0.42), and the difference was not statistically significant (*P* > 0.05) (Table 5). Three months after the operation, IKDC score (73.64 ± 4.46) and Lysholm score (74.76 ± 3.82) in the study group were significantly better than those in the control group (64.84 ± 2.89 and 64.84 ± 2.89), with statistical significance (*P* < 0.001). IKDC score (85.21 ± 2.37) and Lysholm score (85.30 ± 1.99) in the study group were significantly better than those in the control group (77.66 ± 2.82 and 77.53 ± 2.33) 6 months after the operation (*P* < 0.001). 12 months after the operation, IKDC score (90.36 ± 2.07) in the study group was better than that in the control group (88.59 ± 3.20), and the difference was statistically significant (*P* = 0.01). Lysholm score (89.94 ± 2.03) in the study group was better than that in the control group (89.03 ± 1.79), and the difference was not statistically significant (*P* = 0.06) (Tables 5 and 6).

## 4. Discussion

Rehabilitation training after ACL reconstruction is very important. A reasonable and effective rehabilitation plan can help to promote graft healing after ACL reconstruction and restore normal function of the knee joint [7]. However, there is still a great controversy about the rehabilitation training after ACL reconstruction. Previous studies [9, 10] have shown that the transplanted tendon implanted in the knee needs to undergo necrosis, revascularization, crawling replacement of collagen fiber, and molding, and it also takes a long time to form new ligaments with similar ACL characteristics. Orio [11] believed that it would take at least 12 weeks for complete healing of graft and bone tunnel. Therefore, early rehabilitation training programs can relatively shorten the postoperative braking time of patients, but the knee joint adhesion, muscle atrophy, and a long rehabilitation process brought by conservative rehabilitation programs have gradually attracted people's attention. In recent years, early intervention rehabilitation training after ACL reconstruction has been increasingly recognized by scholars at home and abroad [8, 12, 13]. Shelbourne et al. [14] first proposed that full weight bearing and unlimited joint movement could be achieved on the first postoperative day. Yohei et al. [15] found in their study that the strength of graft after fixation for 3 days and 2 weeks was similar, and early movement could be carried out after the operation. Some scholars [16] believe that early weight-bearing exercise after the operation can promote the recovery of early function. Animal studies [17] have shown that early continuous passive joint activity after ACL reconstruction is helpful to create a good microenvironment in the joint and can effectively reduce the risks of traumatic arthritis after ACL reconstruction. Conversely, early immobilization can lead to the development of traumatic arthritis. According to the American College of Sports Medicine, “exercise is medicine” and exercise has anti-inflammatory effects [18, 19]. Appropriate early exercise can reduce joint inflammation and promote cartilage regeneration. Rodeo et al. [20] also believed that appropriate small postoperative weight bearing could promote tendon and bone healing in the early stage. Wen et al. [21] reported that early weight-bearing exercise has retained the original physiological characteristics of articular cartilage and improved functional activities. However, the negative impact of radical rehabilitation training on the recovery of knee joint is also frequently reported. Previous studies have shown that [8, 22–24] radical rehabilitation training has problems such as joint swelling, reconstruction ligament relaxation, bone tunnel enlargement, ligament refracture, and knee instability. Packer et al. [5, 6, 25] believed that excessive weight bearing should not be carried out after reconstruction, and excessive tension of the tendon graft should be avoided after reconstruction. If excessive weight bearing is carried out after reconstruction, it will cause adverse effects on tendon and bone healing. In view of the current controversies, it is urgent to explore a safe, reasonable, and rapid rehabilitation program.

In this study, all patients were treated with the newly created “triangle” braided tensioning line in our department for anatomical reconstruction of ACL. With the protection of ETHIBOND thread in the graft tendon, it can share the bad traction of the graft tendon to the maximum extent so that the graft tendon will not be elongated or only slightly elongated. As can be seen from the results of KT-1000 measurement of forward stability of knee joint, patients using the rapid rehabilitation program in the study group have no obvious relaxation or elongation of the reconstructed ACL after the joint activity training and weight bearing in advance, which also indicates that the rapid rehabilitation program in the study group in this study is safe. In the evaluation of knee function, IKDC score (Table 5) and Lysholm score (Table 6) in the study group at 3 and 6 months were better than those in the control group, which indicated that the knee function of patients in the study group has a faster recovery within 6 months after the operation. As can be seen from the postoperative knee curvature of the patients (Table 7), the rapid rehabilitation program in this study can speed up the recovery of the patient's knee motion.

In conclusion, arthroscopic ACL anatomical reconstruction and tensioning technique combined with rapid rehabilitation therapy can significantly shorten the patient's rehabilitation cycle, not increase the patient's later rehabilitation risk, save medical resources, reduce the patient's economic burden, and improve patient satisfaction.

## Figures and Tables

**Table 1:** Preoperative age, BMI, and time from injury to operation comparison between 2 groups.

Group	Age (years old)	BMI (kg/m^2^)	Time from injury to operation
Study (*n* = 33)	29.06 ± 6.96	22.67 ± 2.51	6.70 ± 3.82
Control (*n* = 32)	29.09 ± 6.48	22.88 ± 1.96	6.53 ± 3.91
*T* value	-0.02	-0.37	0.17
*P* value	0.98	0.71	0.86

**Table 2:** Gender, affected side, and complication of injuries comparison between 2 groups.

Group	Gender		Affected side		Combined with meniscus injury	
	Male	Female	Left	Right	Yes	No
Study (*n* = 33)	20 (60.6%)	13 (39.4%)	18 (54.5%)	15 (45.5%)	22 (66.7%)	11 (33.3%)
Control (*n* = 32)	20 (62.5%)	12 (37.5)	15 (46.9%)	17 (53.1%)	24 (75.0%)	8 (25.0%)
*X* ^2^ value	0.025		0.38		0.55	
*P* value	0.88		0.54		0.46	

**Table 3:** Rehabilitation schedule.

Content						
Time (after the operation)	Weight bearing Walking		Bend knee practice (degree)		Free activities Range with brace (degree)	
	Study	Control	Study	Control	Study	Control
0 days	×	×	×	×	0	0
1-3 days	Partial	Without	45	0	0	0
4-7 days	Partial	Without	90	0	0	0
1-2 weeks	Full	Partial	100	45	30	0
3-4 weeks	Full	Partial	120	90	45	0
4-5 weeks	Full	Partial	125	95	60	30
5-6 weeks	Full	Partial	135	100	75	60
6-7 weeks	Full	Partial	Passive knee bend-normal	110	90	60
7-8 weeks	Full	Full	Passive knee bend-normal	120	100	70
8-9 weeks	Full	Full	Active bend and knee extension-normal	125	Open	90
9-10 weeks	Full	Full	Active bend and knee extension-normal	130	Open	100
10-11 weeks	Full	Full	Active bend and knee extension-normal	Passive knee bend-normal	Remove	120
11-12 weeks	Full	Full	Normal	Passive knee bend-normal	Remove	Remove
3-6 months	Full	Full	Normal	Normal	Remove	Remove

**Table 4:** KT-1000 comparison before and after the operation in 2 groups (mm, *x* ± *s*).

Group	Before	3 months after	6 months after	12 months after	*F* value	*P* value
Study (*n* = 33)	7.18 ± 0.58	2.00 ± 0.00	2.03 ± 0.17	2.09 ± 0.29	1911.57	<0.001
Control (*n* = 32)	7.13 ± 0.42	2.03 ± 0.18	2.03 ± 0.18	2.13 ± 0.42	1965.73	<0.001
*T* value	0.45	-1.00	-0.022	-0.38		
*P* value	0.65	0.33	0.98	0.71		

**Table 5:** IKDC score comparison 3, 6, and 12 months before and after the operation in 2 groups.

Group	Before	3 months after	6 months after	12 months after	*F* value	*P* value
Study (*n* = 33)	49.67 ± 4.93	83.64 ± 4.46	95.21 ± 2.37	99.36 ± 2.07	800.51	<0.001
Control (*n* = 32)	50.88 ± 4.78	64.84 ± 2.89	77.66 ± 2.82	88.59 ± 3.20	687.60	<0.001
*T* value	-1.00	9.47	11.70	2.65		
*P* value	0.32	<0.001	<0.001	0.01		

**Table 6:** Lysholm score comparison 3, 6, and 12 months before and after the operation in 2 groups.

Group	Before	3 months after	6 months after	12 months after	*F* value	*P* value
Study (*n* = 33)	50.58 ± 4.78	84.76 ± 3.82	95.30 ± 1.99	97.94 ± 2.03	895.97	<0.001
Control (*n* = 32)	51.25 ± 4.64	65.09 ± 2.76	77.53 ± 2.33	89.03 ± 1.79	896.09	<0.001
*T* value	-0.58	11.66	14.48	1.91		
*P* value	0.57	<0.001	<0.001	0.06		

**Table 7:** Knee curvature comparison 2, 4, and 8 weeks before and after the operation in 2 groups.

Group	Before	2 weeks after	4 weeks after	8 weeks after	*F* value	*P* value
Study (*n* = 33)	76.18 ± 13.26	88.48 ± 9.35	101.36 ± 6.23	125.85 ± 10.29	145.96	<0.001
Control (*n* = 32)	75.47 ± 13.30	65.03 ± 4.03	91.56 ± 8.41	114.34 ± 11.81	146.39	<0.001
*T* value	-0.22	-13.20	-5.35	-4.19		
*P* value	0.83	<0.001	<0.001	<0.001		

## Data Availability

We confirm that all data were included in our manuscript.
